# Retrograde transport pathways utilised by viruses and protein toxins

**DOI:** 10.1186/1743-422X-3-26

**Published:** 2006-04-07

**Authors:** Robert A Spooner, Daniel C Smith, Andrew J Easton, Lynne M Roberts, J Michael Lord

**Affiliations:** 1 Department of Biological Sciences, University of Warwick, Coventry, CV4 7AL, UK

## Abstract

A model has been presented for retrograde transport of certain toxins and viruses from the cell surface to the ER that suggests an obligatory interaction with a glycolipid receptor at the cell surface. Here we review studies on the ER trafficking cholera toxin, Shiga and Shiga-like toxins, Pseudomonas exotoxin A and ricin, and compare the retrograde routes followed by these protein toxins to those of the ER trafficking SV40 and polyoma viruses. We conclude that there is in fact no obligatory requirement for a glycolipid receptor, nor even with a protein receptor in a lipid-rich environment. Emerging data suggests instead that there is no common pathway utilised for retrograde transport by all of these pathogens, the choice of route being determined by the particular receptor utilised.

## Introduction

A model for retrograde transport of ER-trafficking toxins and viruses from the cell surface to the ER suggests an obligatory interaction with a glycolipid receptor at the cell surface (1).

The bacterial and plant protein toxins that disrupt mammalian cell signalling, cytoskeletal assembly, vesicular trafficking or protein synthesis have cytosolic molecular targets, so at least a portion of the toxin must cross a cellular membrane.

In some cases this is achieved by piercing a biological membrane. This can be the plasma membrane (pertussis adenylate cyclase toxin from *Bordetella pertussis *[2], α enterotoxin from *Staphylococcus aureus *[3], and aerolysin from *Aeromonas hydrophila *[4,5]) or, after endocytosis, the endosomal membrane (diphtheria, anthrax, and botulinum toxins. [6-8]).

Cholera toxin [9,10], Shiga and the very closely related Shiga-like toxins (STx family) [11], *Pseudomonas *exotoxin A (PEx) [12] and the plant toxin ricin [13] seem unable to disrupt cellular membranes directly. After binding their respective receptors at the cell surface, all travel from the cell surface to the endoplasmic reticulum (ER) [14-17], presumably to take advantage of a pre-existing cytosolic entry mechanism. The toxic portions of all these ER-trafficking toxins have unusually low lysine contents so they should be poor substrates for ubiquitination and subsequent proteasomal degradation in the cytosol. Recognition of this led to the proposal that these toxic subunits somehow subvert the ERAD (ER-associated protein degradation) pathway [18], which is the process by which terminally misfolded proteins in the ER lumen are sorted and exported to the cytosol for destruction. Seen in his light, the low lysine complement of these toxins would permit avoidance of degradation, the ultimate fate of normal ERAD substrates. These ER trafficking proteins have thus become tools for probing ERAD and retrograde trafficking pathways.

A number of enveloped viruses such as HIV are able to fuse directly with the host cell plasma membrane to facilitate entry of viral components into the cytosol. Other enveloped viruses such as influenza and non-enveloped viruses such as adenovirus enter the target cell by receptor-mediated endocytosis through clathrin-coated pits. Subsequently, these traffic via the late endosome/lysosome pathway, where they are dismantled prior to endosomal escape. For influenza virus and other enveloped viruses, nucleocapsid delivery to the cytosol requires the low pH environment of the endosome to trigger exposure of a hydrophobic peptide buried within the virus fusion protein, which then stimulates fusion of the viral and endosomal membranes [19]. There is a clear parallel here with diphtheria toxin, where the low pH of the endosome triggers a conformational change in the toxin, permitting engagement of previously occluded tryptophan residues with the endosomal membrane [20]. Exposure of cells to bafilomycin A, an inhibitor of the vacuolar-type H(+)-ATPase responsible for acidifying endosomes, protects them from infection with influenza [21] and from the toxic effects of diphtheria toxin [22].

Strikingly, for productive infection of the non-enveloped viruses simian virus 40 (SV40) and Polyomavirus (Py), there is demonstrable receptor-mediated but clathrin-independent, caveolae-dependent endocytosis followed by obligatory trafficking to the ER. The details of the process(es) by which non-enveloped viruses enter the cytoplasm are currently not well clarified.

Overall, the sites of cytosolic entry of viruses mirror those of protein toxins. This raises the following questions – do toxins and viruses that depend upon retrograde trafficking follow common routes? Are the membrane-breaching mechanisms similar, because they are defined by the nature of the membrane to be traversed, rather than the nature of the virus or toxin? If so, can retrograde-trafficking toxins be used as probes of pathways utilised by some viruses?

Here we review studies that define the molecular mechanisms for retrograde transport of protein toxins to the cytosol, and compare these to known requirements for SV40 and Py viral trafficking and cytosolic entry. Where possible, we base our conclusions on routes that are shown to be productive (for cytotoxicity or infection), since indirect fluorescence localisation may also identify trafficking routes that are non-productive: for example, only a small proportion (~5%) of the ricin that binds a cell traffics (productively) via the trans-Golgi network (TGN), with the remainder directed towards (non-productive) recycling or degradative routes [23].

### ER-trafficking toxin structure and function

Each of the ER-trafficking toxins CTx, STx, PEx and ricin has a catalytic (toxic) A chain associated with either one (PEx and ricin) or five (CTx and STx) cell binding B chains. All are synthesised in non-toxic pro-form, and are subsequently activated by proteolytic cleavage. This releases the A subunit from its A-B precursor (PEx and ricin) or separates a precursor A polypeptide into A1- and A2-chains (CTx and STx). The cleaved products remain disulphide bonded in the mature toxin.

CTx A chain is an ADP-ribosyltransferase that modifies the heterotrimeric G protein Gs-α to activate adenylyl cyclase [24] inducing intestinal chloride secretion, which leads to the massive secretory diarrhoea associated with cholera [25]. At the C-terminus of the CTx A chain is a KDEL ER retention motif, suggesting that the toxin can interact with the KDEL receptor. This receptor recycles between the TGN, Golgi cisternae and the ER, scavenging itinerant soluble ER components and returning them to the ER.

The STx A-subunit and ricin A chain (RTA) are RNA *N*-glycosidases that remove a conserved adenine residue from 28S rRNA [26,27]. This adenine forms part of a motif that is the site of interaction with the EF-2 ternary complex, so intoxication results in cessation of protein synthesis, and, ultimately, cell death [28].

The A chain of PEx ADP-ribosylates elongation factor 2 [12], preventing protein synthesis and leading to cell death. The C-terminus of its A chain contains a KDEL-like sequence.

### From the cell surface to the ER

#### Surface binding and cell entry

**Figure 1:**
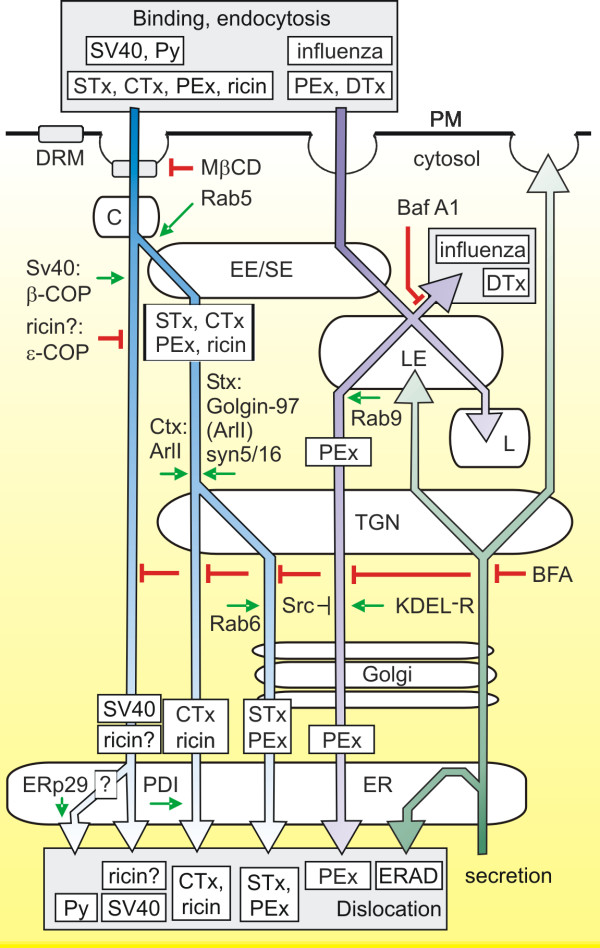
*Generalised simplified retrograde routes available to ER trafficking toxins and viruses*. Association of the toxin/receptor complex or virus/receptor complex with a receptor in detergent resistant membrane microdomains (DRM) facilitates uptake in caveosomes (C) or transport from early/sorting endosomes (EE/SE) to the TGN, directing a proportion of the toxin or virus away from the late endosome (LE)/lysosome (L) pathway and subsequent destruction. A clear exception is Pseudomonas exotoxin A, which can also utilise a LE to TGN pathway to avoid lysosomal destruction. For toxins, transport from the TGN to the ER may proceed via the Golgi stack or may be direct: for SV40 and Py, ER transport appears to proceed directly from caveosomes.

ER-trafficking toxins bind membrane receptors via their B chain(s) and then enter the cell by endocytosis (Figure 1) [15,16].

CTx B chain binds a membrane glycolipid, the ganglioside GM1 [29,30] with up to 5 gangliosides being bound per holotoxin molecule, contributing to a theoretically high avidity of binding. The STx family members are also glycolipid-specific, interacting with the trisaccharide domain of globotriaosylceramide (Gb_3_/CD77) [31-33]. Each STx B subunit has 3 receptor binding sites, so the potential avidity of binding is very high [34]. Cross-linking of Gb3 promotes toxin recruitment into cell surface lipid rafts prior to cell entry [35-37], and also stimulates intracellular signalling cascades that result in cytoskeletal remodelling [38-41]. Thus, binding of STx may stimulate and control its own endocytosis.

Ricin B chain (RTB) is a lectin that binds exposed β1-4 linked galactosides [42]. Cell binding is highly promiscuous because a wide range of cell-surface glycoproteins and glycolipids display these galactosides. Ricin receptors appear to be largely proteinaceous in nature [43]. However, the combination of high number of binding sites per cell and the low affinity of binding [44,45] means that, to date, no specific ricin receptors have been defined. Since RTB has two galactose-binding sites, there is potential for cross-linking of receptors by toxin challenge, with subsequent establishment of signalling cascades.

PEx binds a membrane protein, the α_2_-macroglobulin receptor/low-density lipoprotein receptor-related protein [46]. In contrast to all the other ER-trafficking toxins known, its crystal structure gives no suggestion of high valency binding to its receptor [47].

Binding of these ER-trafficking toxins to their respective receptors is required for endocytosis, which occurs by multiple mechanisms, delivering the toxins to the early and recycling endosomal (EE/RE) compartment [48]. During this early entry process, if required, activation of the toxin by furin cleavage will occur. CTx and ricin are pre-activated. CTx is activated by mammalian intestinal enzymes prior to target cell binding, and ricin activation occurs in the seeds of the producing plant, *Ricinus communis*. In the EE/RE environment the A subunit of STx is cleaved into disulphide-linked 29 kDa A1 and 3 kDa A2 chains and the PEx proenzyme is cleaved to produce an N-terminal B chain of 28 kDa disulphide-linked to a C-terminal A chain of 37 kDa.

Like CTx and STx, SV40 and Py bind glycolipid receptors in the plasma membrane of host cell [49]. SV40 binds the ganglioside GM1 and Py binds the gangliosides and GD1a and GT1b.

In some cells, SV40 enters via caveolae and infection is inhibited by caveolar disrupting agents such as the cholesterol-binding methyl-ß-cyclodextrin (MßCD) and the cholesterol depleting nystatin [50-53]. Py enters at least some mouse cells by a pathway that depends neither on caveolae nor on clathrin [54] and infection of primary baby mouse kidney epithelial cells and established murine fibroblasts by Py is insensitive to disruption of caveolar function by treatment with either MßCD or nystatin. These findings strongly suggest that uptake of these two related viruses in the same cells follows different pathways. These results stand in contrast to those with other mouse cell lines in which Py infectivity was found to be significantly inhibited by treatment with MßCD [55]. Different host cells may therefore differ in their susceptibilities to different cholesterol-binding drugs used to assess caveolar function and virus uptake. It is also possible that the same virus may utilize different cellular pathways for uptake indifferent cells. Indeed, in a caveolin-1 (cav-1)-deficient cell line (human hepatoma 7) and embryonic fibroblasts from a cav-1 knockout mouse, SV40 exploits an alternative, cav-1-independent pathway and this alternative pathway is also available in wild-type embryonic fibroblasts [56]. Internalization here is cholesterol and tyrosine kinase dependent but independent of clathrin, dynamin II, and ARF6. The viruses were internalized in small vesicles and transported to membrane-bound, neutral pH organelles similar to caveosomes but lacking the caveolar markers cav-1 and -2. They were next transferred by microtubule-dependent vesicular transport to the ER, a step required for infectivity.

#### From the endosomes to the TGN

At least two retrograde pathways proceed from endosomes to the TGN (Figure 1); [57-60]. One is dependent on the small GTPase Rab9 and operates from late endosomes (LE) [61]. The other is Rab9-independent and leads from an early endosomal (EE) compartment [17] These pathways also depend on separate vesicle- and target-organelle-soluble *N*-ethylmaleimide-sensitive fusion attachment protein receptor complexes (v-SNAREs and t-SNAREs, respectively) to achieve fusion of intracellular vesicles.

For the glycolipid-specific CTx and STx family, transport from early endosomes to the TGN depends on lipid transport and requires a critical association with detergent resistant membrane microdomains (DRM). STx retrograde transport depends on the TGN t-SNARES syntaxins 5 and 16 [62], and on the Arl1 GTPase effector Golgin-97 [63]. CTx enters cells in vesicles containing the early endosome marker Rab5 but lacking lysosomal markers [64]. Subsequently, it accumulates in a discrete population of endosomes lacking classical EE markers *en route *to the TGN [65]. From the early endosome to the TGN, CTx traffics in Arl1 dependent vesicles [63] indistinguishable from those that carry STx [66]. Thus, like STx, productive routing of CTx is thought to avoid the late endosomes and lysosomes in a Rab9-independent manner.

Association with lipid-rich plasma membrane domains and subsequent Rab5 dependent trafficking into a cell seem to be common entry strategies, even appearing to be mandatory for productive HIV-1 infections in non-CD4+ cells [67]. A clear exception is PEx. Whilst a proportion of cell bound PEx can traffic in this manner in HeLa cells, the majority enters cells independently of DRM association and is sorted at the early/recycling endosome compartments in a non-lipid dependent manner [68], subsequently trafficking to the TGN in a Rab9-dependent manner from late endosomes. In murine Swiss 3T3 cells, PEx appears to be constrained to this Rab9-dependent route. Ricin receptors are predominantly proteinaceous [43], so ricin might be expected to follow a similar route, but in fact its transport is Rab9-independent [69] and sensitive to MβCD [70], and some enters cells in Rab5-positive vesicles [71], so at least a proportion of ricin trafficking appears to be CTx-like and STx-like from the cell surface to the TGN.

#### From the TGN to the ER

At least two routes have been described for protein toxin travel from the TGN to ER, but recent work with toxins suggests a third very poorly characterised route exists (Figure 1).

In the first, there is a critical dependence on binding KDEL receptors which cycle between the TGN and the ER via the Golgi cisternae [72] in a COP1-dependent manner and which typify retrograde transport in the classic secretory pathway [73,74]. PEx trafficking down the Rab9-dependent route needs to disengage from its primary receptor and then associate with KDEL receptors. Since the A chain of PEx terminates in a KDEL-like sequence, it is thought that the KDEL-receptor then delivers PEx from the TGN into the lumen of the ER [68,75-78]. This pathway appears to be very important for PEx as PEx transport is accelerated after inhibition or genetic ablation of the tyrosine kinase Src [79], which regulates KDEL-receptor distribution.

In a second TGN to ER pathway, the lipid-sorted pathway utilised by STx traffics from the TGN to the ER in a COP-I independent manner, in a manner controlled by Rab6 [59,80-82]. PEx bound to DRM at the cell surface, which enters the cells in a Rab9-independent manner, can also traffic via this route [68].

In the third pathway, CTx moves directly from the TGN to the ER without passing through the Golgi cisternae [83] and therefore independently of COP-I vesicles and the KDEL receptor. What, then, is the function of the KDEL sequence at the C-terminus of the A2 chain of CTx? It is proposed that this prevents CTx delivered by lipid receptors moving anterograde from the ER to the *cis *Golgi: thus the KDEL sequence acts as a recycling accumulator, promoting high concentrations of CTx in the ER for subsequent dislocation of the A1-chain to the cytosol.

Ricin's promiscuous binding and the lack of defined receptors lead to poor knowledge of events between the TGN and the ER. Ricin lacks a KDEL retention sequence, but can interact with the chaperone calreticulin in the Golgi complex. Calreticulin has a KDEL-motif, and may traffic to the ER in the COP-I dependent pathway by binding the KDEL-receptor when bound to ricin [84], although this is unlikely to be a major route, since calreticulin-deficient cells remain equally sensitive to ricin. Ricin can also bind glycolipids that contain terminal galactose, and so a proportion may follow lipid sorting signals. The TGN-to-ER pathways exploited by ricin remain unclear however, since RTA can kill cells inhibited simultaneously in both the classical COP1-dependent and Rab6-dependent pathways [85], suggesting that ricin can also bypass the Golgi stack in a CTx-like manner.

#### Bypassing the TGN and Golgi stack

Details of the pathways taken by SV40 and Py to reach the ER are still under investigation. After infection Py can be co-localized with the ER luminal protein BiP [86]. SV40 infection is strongly inhibited by expression of GTP-restricted Arf1 and Sar1 mutants and by microinjection of antibodies to β-COP, suggesting that infection requires COP-I-dependent transport steps for successful infection [87]. Subsequent transport to the ER is sensitive to the fungal metabolite brefeldin A (BFA) [88] which, in cells with a BFA-sensitive Golgi apparatus, causes fusion of Golgi and ER membranes, and thus disrupts both anterograde and retrograde trafficking between these organelles. These results appear to implicate the Golgi apparatus as a staging post for the viruses *en route *to the ER. However, although SV40 co-localizes with β-COP it does not co-localise with Golgin-97 [89], which at steady state resides in the TGN [90,91]. β-COP is also a marker of caveosomes [92] as well as the Golgi [93-96]. The BFA sensitive retrograde step is thus likely to reflect blocking of caveosomal/endosomal escape, rather than a requirement for the Golgi, since BFA treatment also results in fusion of endosomal, lysosomal and TGN membranes [97]. Thus the caveosome appears to be a BFA-sensitive sorting organelle from which at least two distinct routes emerge, separating the retrograde trafficking of CTx and SV40 [98,99] (Figure 1). The former proceeds to the TGN via the EE, whilst the virus traffics directly from the caveosomal early sorting vesicle to the ER thereby bypassing the TGN and the Golgi stack. Curiously, unusual ricin trafficking directly from an early sorting vehicle to the ER can be induced in CHO cells carrying a temperature sensitive ε-COP under conditions where ε-COP is inactivated [100]: the promiscuity of ricin binding may allow it to access an SV40-like retrograde route when its normal retrograde routes are unavailable.

#### The ER provides necessary unfolding activities

The ER is a site from which misfolded proteins can be dislocated via the Sec61 translocon to the cytosol in the process termed ERAD. At least one correctly folded protein, (calreticulin, normally regarded as an ER resident), can also be unfolded to enter the cytosol from the ER, via the translocon, and refolds in the cytosol to avoid degradation [101]. Since the translocon has a narrow pore [102,103], there is thought to be a requirement for unfolding, and this requires protein chaperones, an abundance of which reside in the ER lumen. Presumably toxins and viruses that traffic to the ER do so to take advantage of these pre-existing unfolding and cytosolic entry mechanisms.

Mature, activated (proteolytically cleaved) toxins arriving in the ER have their A and B or their A1- and A2-chains tethered by a disulphide bond. Ricin holotoxin is inactive against free ribosomes *in vitro*, because the B chain hinders A chain catalytic activity [104], so reduction of the subunits is a requisite for cytotoxicity. This is assumed to be the case for the other ER-trafficking toxins.

ER-delivered CTx is a substrate for the ER chaperone protein disulphide isomerase (PDI), which dissociates the A1-chain from the rest of the toxin [105] and then reduced PDI unfolds the released A1-chain. At the ER membrane, the ER oxidase ERO1 catalyzes the re-oxidation of PDI, releasing the unfolded A1-chain to the dislocation machinery [106]. The ER chaperone BiP may also participate in unfolding CTx A1-chain [107]. PDI may also reduce PEx [108], and it is assumed that PDI, or some other reducing agent, is also responsible for separating the A1- and A2-chains of STx.

Reduced PDI also reduces ricin into constituent A and B subunits [45], with a role for thioredoxin reductase as an agent for reducing PDI [109]. Liberated RTA interacts with negatively-charged lipids, undergoing structural changes and promoting membrane instability [110]. ER chaperones might also recognize newly exposed RTA domains to catalyze unfolding reactions. It is thought that partially unfolded RTA now masquerades as an ERAD substrate, interacting with ER components that direct them from the ER to the cytosol. Evidence for a functional correlation between ERAD and sensitivity to ER-directed toxins has been provided by mutant cell lines that display either decreased or increased ERAD activities [111,112]. Thus PDI-catalysed unfolding of CTx and partial unfolding of RTA at a lipid membrane may allow their recognition as misfolded substrates for ER components normally associated with ERAD. Consistent with this notion, STx interacts with the ER luminal chaperone HEDJ/ERdj3, in a complex that includes the ER chaperones BiP and GRP94 and also the Sec61 translocon [113].

The membrane penetration of non-enveloped ER-trafficking viruses is a poorly understood process. Strikingly, though, a requirement for interaction with an ER oxidoreductase related to PDI has recently been described [114], suggesting that interactions with ER chaperones are as important for ER-trafficking viruses as they are for ER-trafficking toxins. A PDI-like protein, ERp29, triggers a conformational change in the Py protein VPI, partially unfolding it to expose its C-terminal arm. ERp29-modified VP1 can interact with liposomes, and by extension, probably therefore with the ER membrane, in preparation for membrane penetration. In support of this, expression of the dominant-negative N terminal domain of ERp29 decreases Py infection, indicating ERp29 facilitates viral infection.

## Dislocation

After a protein is identified as an ERAD substrate, it is exported from the ER to the cytosol for destruction. Experiments showing mammalian ER export of dislocated MHC class I heavy chains mediated by the product of the cytomegalovirus US2 gene, [115,116] and studies with specific yeast mutants [117,118], first suggested that export in both systems involved the Sec61 translocon in a reversal of the process by which nascent secretory proteins are delivered into the ER lumen. Both CTx and ricin can be co-immunoprecipitated with sec61 [119,120]. There is also evidence that PEx can use the Sec61 complex for dislocation [121]. For STx, interactions of the toxin with ER chaperones in a complex that includes the Sec61 translocon suggest that this toxin also utilises the translocon for egress from the ER [113].

The driving force for ER dislocation of any protein toxin remains unknown, but it is likely that this is supplied by a cytosolic motor. Almost all terminally misfolded proteins known to be dislocated are poly-ubiquitinated on lysine residues, but a mutant CTx A1 chain with its N-terminus chemically blocked and all lysines mutated to arginine [122] and a ricin holotoxin reconstituted from plant-derived RTB and a recombinant RTA lacking all lysines [123] remain fully toxic. The AAA-ATPase p97 and its adaptor molecules Ufd1 and Npl4 are involved in dislocation of some ERAD substrates and it seems reasonable to suggest that they may be involved in toxin dislocation, but to date, the data conflict [124,125].

How the membrane-embedded Py reaches the cytosol is currently unknown. The low cholesterol concentration of the ER membrane makes it passively permeable to small molecules which are unable to cross the plasma membrane or the lysosomal and trans-Golgi membranes [126]. This general property could allow the virus-membrane interaction to induce holes in the bilayer by disrupting the phospholipid organization, thereby enabling the virus to egress the ER. Cytosolic chaperones could bind to the exposed hydrophobic regions of Py on the cytosolic surface of the ER membrane and extract the virus into the cytosol, similar to the manner proposed for dislocating toxins through the ER translocon. Overall it is clear that the motor(s) required for dislocation of protein toxin subunits and viruses remain a mystery.

## Conclusion

Figure 1 depicts generalised retrograde transport routes, but of necessity, shows a degree of over-simplification. Thus, SV40 transport is shown to proceed from caveosomes, although this is not obligatory for infection [56,99] so there may be further sorting in early endosomes; ricin and CTx transport is depicted as STx-like from early endosomes to the TGN, although there may be multiple routes; and CTx and ricin are depicted as following a single route from the TGN to the ER, but this is poorly characterised, without known markers. Furthermore, there are cell-type differences in entry of CTx [127], PEx, [68] and SV40. Also, entry route may alter at different concentrations of virus or toxin, and molecular disturbance of one trafficking pathway may induce others. Finally, we have tried to limit this compiled figure to routes known to be productive for viral infection or intoxication. For example, treatment of cells with MßCD has very little effect on total ricin endocytosis [128], but strongly attenuates cytotoxicity [70] suggesting that the majority of endocytosed ricin is recycled or degraded.

Nevertheless, the Figure points out that ligands with a common receptor (eg. SV40 and CTx) can reach the ER by different routes, and that a toxin with a single known protein receptor (PEx) can access different routes dictated by cell-surface binding events [68]. Despite observations of co-localisation of CTx and SV40 in caveolae [89,129,130], a common Rab5-dependent trafficking of CTx, Stx, Py and SV40 from such structures to early endosomes [66,99] and a proposal that interaction with detergent resistant membranes is required for ER transport [1], we suggest that there are very few aspects in common between the retrograde routes available to the viruses Py and Sv40 and ER-trafficking toxins. It is more likely that rather than all being constrained to one retrograde route, each virus or toxin traffics in a manner determined by its own peculiar interaction with receptor. However, the site of cytosolic entry provides insights into common mechanisms. Low pH-stimulated conformational changes in influenza proteins and diphtheria toxin are appropriate for endosomal escape. For the ER trafficking viruses and toxins, then, presumably common interactions are made, defined not by the nature of the ER trafficking entity, but the nature of the ER lumen. Strikingly, members of the ER oxidoreductase family are seen to be important. These promote reduction of toxin subunits, but may also reductively activate Py VP1 since the effects of ERp29 are amplified in reducing conditions that could mimic PDI action [114]. Furthermore members of this family are also implicated in stimulating conformational changes in both toxins and viral proteins. To date, details of ER escape mechanisms are poorly understood, beyond a likely requirement for the Sec61 translocon for toxins, but we fully expect dislocation motors for both toxins and viruses to show strong similarities.
